# A Participatory Approach in Assessing the Knowledge, Attitude, and Practices (KAP) of Stakeholders and Livestock Owners about Ticks and Tick-Borne Diseases from Sindh, Pakistan

**DOI:** 10.3390/pathogens12060800

**Published:** 2023-06-03

**Authors:** Mahvish Rajput, Muhammad Sohail Sajid, Muhammad Imran, Muhammad Tariq Javed, Olivier Andre Sparagano

**Affiliations:** 1 Department of Parasitology, University of Agriculture, Faisalabad 38040, Pakistan; drmehvish.aslam@gmail.com (M.R.); imran.asghar@uaf.edu.pk (M.I.); 2 Department of Pathology, University of Agriculture, Faisalabad 38040, Pakistan; mtjaved@uaf.edu.pk; 3 Department of Infectious Diseases and Public health, Jockey Club College of Veterinary Medicine and Life Sciences, City University of Hong Kong, Kowloon, Hong Kong 999077, China

**Keywords:** livestock, ticks, tick-borne diseases, zoonosis, participatory epidemiology

## Abstract

Ticks and tick-borne diseases (TTBDs) are responsible for significant losses in terms of treatment costs, decreased productivity (such as milk and meat), reduced reproductive ability, and financial crisis to livestock owners. In Pakistan, it is crucial to periodically assess the risk of TTBDs and ecological factors, potential causes of acaricidal resistance in tick fauna, and the intensive increase in the spread of TTBDs. Participatory epidemiological approaches are key to assessing the livestock owners’ and stakeholders’ knowledge, attitude, and practices (KAP) about TTBDs. The current study determined the KAP about ticks and tick-borne diseases of respondents from Sindh, Pakistan. A total of 240 respondents were interviewed from different ecological zones: among them, 42.5% (n = 102) of the respondents practiced the manual removal of ticks from animals, while acaricide usage was indicated by 137 respondents (57.0%) as occurring sometimes, 50 (20.8%) monthly, 41 (17.0%) fortnightly, and 12 (5%) weekly, during the peak infestation season. Ticks were 2.6 times [OR = 2.5 (95% Cl = 1.47–4.06)] and viruses were 1.89 times [OR = 188 (95% Cl = 1.09–2.9)] more likely to cause the development of disease in animals than any other pathogen. Despite the appropriate usage of acaricides, the knowledge of participants was inadequate. The findings of this study emphasize the need to take into account identified knowledge gaps and to take the initiative in carrying out appropriate education activities and extension programs to enhance the adoption of effective tick prevention and control strategies.

## 1. Introduction

Pakistan is predominantly an agricultural country, where the agriculture sector plays a critical role in its overall prosperity. Within this sector, livestock is a particularly important subsector, controlling up to over 60.5% of the agriculture value addition and nearly 11% of the country’s GDP. In addition, approximately 35 million people are employed in this sector [1]. Moreover, 70% of Pakistan’s population resides in rural areas, and the majority of them depend on livestock for their livelihood [2]. Pakistan is home to a diverse range of livestock species, including cattle, buffaloes, sheep, goats, camels, horses, and poultry. Ticks (Acari: Ixodidae) are known to play a significant role in the spread of diseases among livestock, humans, and domestic animals worldwide. They are capable of transmitting various micro-organisms such as viruses, bacteria, protozoa, rickettsia, and spirochetes, mechanically or biologically, causing tick-borne diseases (TBDs) [3]. TTBDs are the major cause of financial losses to livestock owners throughout the world [4,5]. The distribution of ticks is affected by various biotic and abiotic factors, and the ideal humidity and temperature ranges of 60–80% and 27–39 °C, respectively, are necessary for their growth and sustainability [6]. Pakistan is geographically located in a subtropical zone (30° N, 70° E) with favorable environmental conditions for tick development and the transmission of TBDs such as babesiosis, anaplasmosis, and theileriosis [7]. The Sindh province in Pakistan is known to have several species of ticks that are potential vectors of various tick-borne diseases [8]. Various ticks were identified from all districts of Sindh and the most commonly infested ticks belonged to genus Hyalomma, followed by Rhipicephalus, Boophilus, and Amblyomma [9]. Therefore, it is crucial to conduct a periodic risk assessment of the TTBDs and ecological factors, identify potential causes of acaricidal resistance in tick fauna, and monitor the spread of TTBDs intensively [10]. 

Participatory epidemiology (PE), an approach involving the active participation of stakeholders, including farmers, veterinarians, and other community members, can be used to improve disease control and management strategies by involving local communities in disease surveillance, diagnosis, and treatment [11]. In the context of TTBDs, participatory epidemiology can help to obtain farmers’ knowledge and attitude about the diseases [12,13]. In the participatory epidemiology study of ticks and TBDs in the livestock of Pakistan, key stakeholders, including farmers, veterinary professionals, and representatives from local government agencies or NGOs, can be involved in the study design and its implementation [14]. Through formal and informal interviews, focus group meetings, and other community outreach activities, the study can be made relevant and useful to local communities [15]. By identifying the various risk factors through interviews and surveys with farmers and other stakeholders, strategies for tick infestation and TBD control, such as improved animal husbandry practices, targeted use of acaricides, and vaccination programs, can be developed and implemented [16]. Overall, participatory epidemiology can help to improve the understanding of ticks and TBDs in livestock in Pakistan, and to develop effective strategies for disease control and management that are tailored to local conditions and community needs.

This study aimed to assess the impact of ticks and TBDs on livestock production in selected districts of the various agro-ecological zones of Sindh, Pakistan using PE methods. In addition, the study aimed to identify current tick and TBD control methods utilized by livestock owners and any regional barriers to their implementation. The gathered information will be useful in developing effective animal health programs to improve livestock productivity and enhance the living standards of livestock owners in Pakistan.

## 2. Materials and Methods

To collect the information about the ticks and TBDs from livestock owners from selected districts of Sindh, Pakistan. The first methodology involved conventional survey methods conducted by trained veterinarians, while the second methodology involved participatory approaches. The participatory approach recognizes the importance of local knowledge and participation in research, and aims to involve all stakeholders in problem solving and disease management. In particular, participatory epidemiology (PE) was used in the evaluation and design of disease management strategies [11].

### 2.1. Study Area

**Figure 1:**
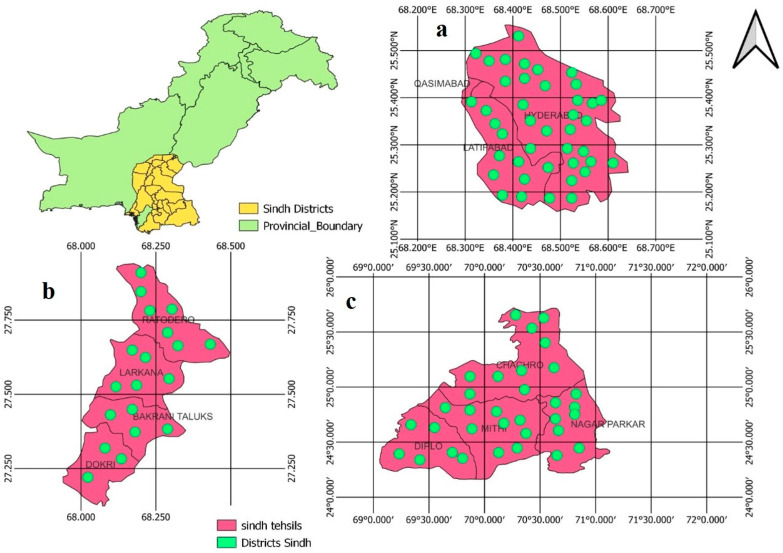
Physical map of Sindh province showing selected districts: (**a**) Hyderabad, (**b**) Larkana, and (**c**) Tharparkar. Maps have been generated using QGIS software.

Sindh, Pakistan is divided into three agro-ecological zones (AEZs) based on factors such as climatographic parameters, land use, physiography, soil type, and climate conditions. To conduct this study, three districts (Hyderabad, Larkana, and Tharparkar) were selected (Figure 1), on the basis of their environmental conditions and their belonging to different AEZs [17]. A cross-sectional study was conducted using triangulation, a participatory epidemiology (PE) method, from December 2022 to February 2023. Respondents were chosen from each district in collaboration with the Livestock Departments and Benazir Bhutto Youth Development Program of Sindh, Pakistan. The Livestock Department of Sindh provided great support during the fieldwork and assisted in identifying suitable respondents for data collection in the selected districts.

### 2.2. Questionnaire Survey

To collect data on various factors related to livestock production and TTBDs, a structured questionnaire consisting of 44 items was designed (Supplementary Materials S1). The questionnaire covered a range of topics such as sociodemographic information, farming practices, housing and feeding types, risk factors, acaricidal practices, and hygienic conditions. The questionnaire was prepared in multiple-choice format following [18], with some modifications and refinement through informal and formal testing procedures. Selected study areas were visited twice a month from Dec 2022 to Feb 2023. 

To ensure the questionnaire was effective in gathering accurate information, it underwent pilot testing [19]. Before administering the questionnaire to the target population, it was reviewed by several researchers who had used similar questionnaires in epidemiological studies. The study areas were visited twice a month from December 2022 to February 2023, and the questionnaire was administered to livestock owners in the selected villages.

### 2.3. Selection of Respondents

To select the respondents for the study, age and livestock ownership were taken into consideration [20]. The majority of respondents were household heads or senior members who lived in the household throughout the year and were responsible for livestock management. Prior to conducting the interviews, the enumerators explained the purpose of the study to the selected respondents and obtained verbal consent for the interview, following the guidelines outlined by Singer [21]. The respondents were informed that their participation was voluntary and they could withdraw from the study at any time. The interviews were conducted in local dialects but recorded in English for accuracy and transcription purposes.

### 2.4. Data Collection and Management

The collected data were carefully reviewed and filtered for completeness before the analysis. A binary outcome variable was created based on the respondents’ knowledge of tick-borne diseases, with a score of 1 indicating sufficient knowledge and 0 indicating inadequate knowledge. The study included livestock owners with ruminant herds of 10 to 50 animals, and the farmers were selected based on operational convenience and willingness to participate [22]. A total of 240 face-to-face interviews were conducted using a structured questionnaire, which was prepared in English but translated into local languages such as Urdu and Sindhi during the interviews to ensure accuracy of responses and to minimize confusion. All the collected data were transferred to MS Excel (Microsoft Excel 2016, Redmond, USA) spreadsheets for statistical analysis.

### 2.5. Statistical Analyses

All the collected data from 240 respondents in the form of questionnaire with record of demographic statues of respondents, knowledge about ticks and tick-borne diseases and their associations with different risk factors, ecological parameters, housing types, feeding systems, attitudes, and practices of farmers were analyzed using multiple logistic regression and odds ratio. All the data were analyzed using SPSS software V25.0 [23]. 

### 2.6. Ethical Approval

This study was conducted with the approval from the Veterinary Institutional Bioethics and Institutional Animal Care and Use Committee (IACUC), University of Agriculture Faisalabad, Vide No. 34989-92 dated 15-12-2022. All activities involving human participants adhered to ethical guidelines for protection of participants’ rights and well-being [24]. After the participants had given their agreement, data were gathered.

## 3. Results

### 3.1. Socio-Demographic Analysis of Farmers of the Sindh Region

The study included 240 participants from three districts of Sindh, Pakistan, with a wide range of educational backgrounds. About 40.4% of the farmers had not attended any school, while 5% were graduates. The majority of the farmers (74.5%) were from rural areas, and the rest were from urban areas. A large proportion (54.1%) of the farmers owned livestock, while 27% were community members, and only 18.75% were veterinarians.

**Figure 2:**
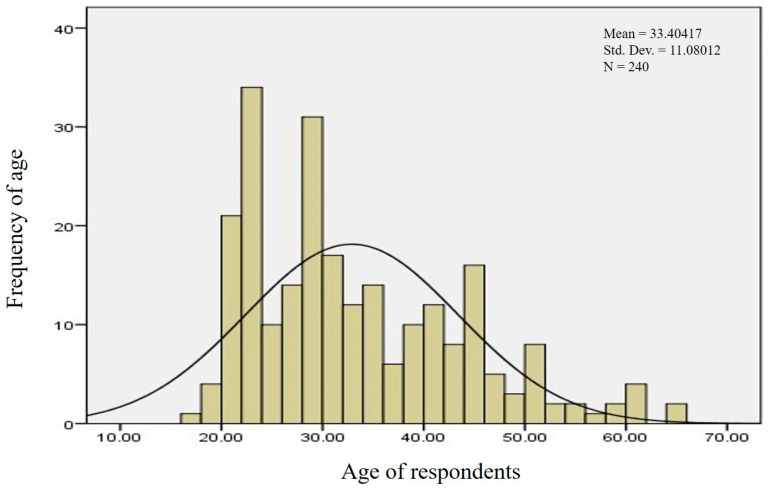
Age of respondents.

Age was a significant variable in this study, with farmers between the ages of 18 and 70 years old included in the analysis. Over half of the farmers (53.3%) were between the ages of 18 and 30, while 40.8% were between the ages of 31 and 50. Only 5.8% were between the ages of 51 and 70. The mean age of respondents in Sindh was found to be 33.40 years, with a standard deviation of 11.080 (Figure 2). Adult-age farmers were observed to be more interested in adopting new tools and technologies to protect their livestock from ticks and tick-borne diseases.

### 3.2. Awareness of TTBDs in Livestock

All participants were interviewed face-to-face and the knowledge of the participants regarding ticks and ticks-borne diseases was gathered. When asked if they had seen ticks, 220 out of 240 farmers (91.6%) answered yes, while only 20 (8.3%) said no. Out of the 220 farmers who had seen ticks, 102 (46.36%) reported observing them on animals, 20 (9.09%) in forests, 59 (26.81%) in agricultural lands, 15 (6.8%) in pastures, and 24 (10.9%) had observed ticks in all the above-mentioned places.

When asked about the prevalence of ticks in different seasons, 114 farmers (47.5%) believed that ticks were more prevalent in warm places, while 14 farmers reported a tick presence even in cold weather. However, 112 (46.6%) farmers claimed to have observed ticks equally in both cold and warm seasons, but the intensity was different from each other. In Pakistan, the summer season is characterized by temperatures ranging from 35 °C to 45 °C, while winter temperatures range from 26 °C to 4 °C. Accordingly, 200 (83.33%) farmers had observed ticks throughout the year, 20 (8.3%) had observed ticks only in the winter season, and another 20 (8.3%) had seen ticks only in the summer season.

Of the respondents, 85 (35.41%) believed that the livestock acquire ticks from the forest. Other respondents believed that the livestock acquire ticks from agriculture land, grazing area, and pasture land. When asked about which age group of the livestock is most severely affected by ticks, the majority of respondents (n = 111, 46.25%) stated that old animals are more susceptible, followed by young animals chosen by 64 respondents (26.66%), adult livestock chosen by 40 (16.66%), heifers chosen by 15 (6.25%), and 10 (4.1%) who responded “Don’t know”.

In terms of breed susceptibility, 209 respondents (87%) stated that native breeds are more resistant to ticks and tick-borne diseases compared to exotic breeds (n = 25, 10.41%), while 6 (2.5%) were unsure. When asked about the most common tick predilection sites on livestock, the neck and groove regions were deemed the most prevalent by 233 (94.7%) and 190 (77.2%) of respondents, respectively. In addition, 113 (47.08%) respondents felt that ticks would remain on the animals’ bodies unless removed, while 111 (45.1%) believed that ticks would drop off after feeding on the animal’s blood. Only 16 (6.6%) respondents reported that they did not know.

**Figure 3:**
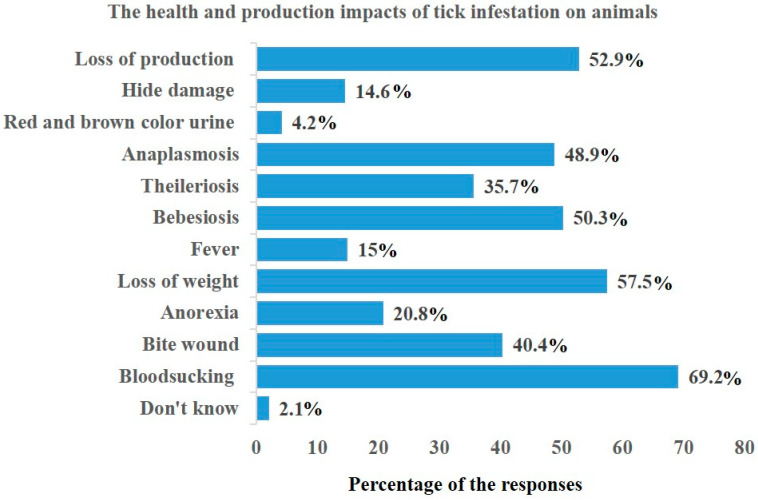
Determination of health and production impacts of tick infestation on animals.

The study found that all the farmers (n = 240, 100%) believed that ticks can transmit diseases to livestock, and that ticks stay on the animals’ bodies until removed. The farmers reported observing various symptoms (listed in Figure 3) in livestock infested with ticks. The most commonly reported symptoms were bloodsucking and anorexia (n = 205, 85.4%), and many respondents provided multiple answers. The analysis of knowledge scores revealed that 118 (49.16%) [95% CI: 45.5–58.4] of the 240 respondents had adequate knowledge about ticks as potential carriers of diseases in both animals and humans.

### 3.3. Attitude of Respondents

To determine the attitude of participants regarding the possible methods of reducing tick infestation, open-ended questions were asked. In the conclusion, names were double-checked with the neighborhood vet or para-vet staff. 

After the information was gathered, participants were asked to score each variable (Table 1) based on how important they believed it to be (i.e., strongly agree, disagree, no opinion, agree, and strongly agree). Based on the limitations’ perceived relevance (ranked from 1 to 5, where 1 is most essential and 5 is least important), this activity generated a list of restrictions (mean: 3, range: 2–4). 

### 3.4. Husbandry Practice and Common Animal Health Problems

Based on our scoring criteria, multiple logistic regression showed that husbandry practice and common animal health problems were the significant variables in the fit model. The results demonstrated that ticks were 2.6 times [OR = 2.5 (95% Cl = 1.47–4.06)] and viruses were 1.89 times [OR = 188 (95% Cl = 1.09–2.9)] more likely to have auspicious effects towards the development of disease in animals than any other pathogen (details listed in Table 2). Respondents who practiced stall feeding were shown to be 1.8 times [OR = 1.8 (95% Cl (1.08–3.43)] more likely to have a favorable attitude than those who practiced mixed livestock rearing.

This analysis included no confounding variables, outliers, or significant observations. The association between livestock keeping and husbandry practices was not statistically significant.

### 3.5. Ticks and Their Zoonotic Importance

**Figure 4:**
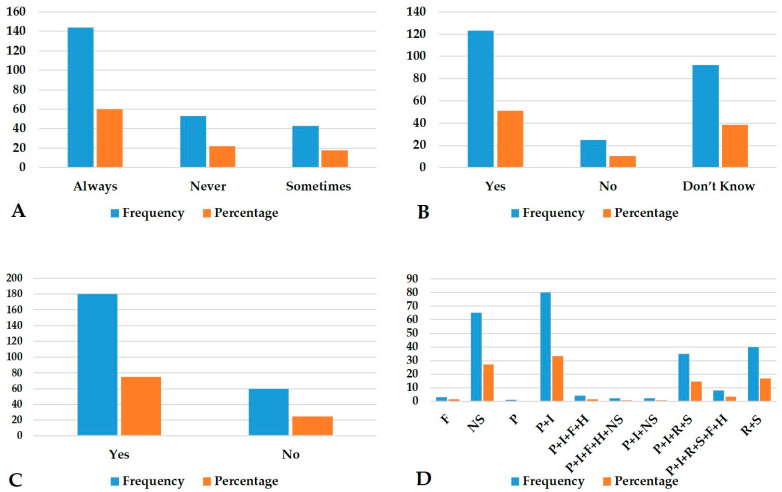
Descriptive analysis of the exposure of respondents and ticks in the farming region of Sindh: (**A**). Checked body for ticks after handling the tick-infested animals; (**B**). Humans can get diseases from the tick bites; (**C**). Bitten by ticks; and (**D**). Clinical symptoms of F = fever; NS = No symptoms; P = Pain; P + I = Pain and irritation; P + I + F + H = Pain, irritation, fever, and headache; P + I + F + H + NS = Pain, irritation, fever, headache, and no symptoms; P + I + NS = Pain, irritation, and no symptoms; P + I + R + S = Pain, irritation, rash, and swelling around bite; P + I + R + S + F + H = Pain, irritation, rash, swelling around bite, fever, and headache; and R + S = Rash and swelling around bite.

Participants were interviewed and the response rate was 100%. The details of human exposure to ticks are presented in Figure 4A–D.

### 3.6. Respondents’ Practice and Management of Ticks and Tick-Borne Diseases

The practices of respondents in rearing livestock were investigated, and the main purpose of rearing livestock in the households of farmers was also estimated. The results showed that 86 (35.83%) farmers reared livestock for family consumption. A large number of farmers (n = 92, 40%) rear animals for income generation through the sale of products and animals, 50 (20.83%) farmers used livestock as a source of manure draft, and 12 (5%) farmers reared animals at home for breeding purposes. The type of livestock shed used by farmers of the Sindh region was also investigated and they reported that 95 (39.58%) respondents used improved sheds with a CGI Sheet, 69 (28.75%) participants used concrete flooring, improved sheds with a CGI sheet, and wooden flooring, and 76 (31.66%) used conventional sheds (built with local materials; open-air tethering). If the livestock shed’s floor was made of concrete, it was then further investigated how often they washed the flooring of the livestock shed. It was intimated that the shed was washed by 52 (21.66%) respondents on a daily basis and 42 (17.5%) respondents washed it on weekly basis, 47 respondents (19.58%) washed it fortnightly, 24 (24.16%) respondents washed it on monthly basis, and 41 (17.08%) respondents claimed that they had never washed the animal sheds. Bedding material was also used in the animal sheds for the provision of protection to the animals from ticks, and it was estimated that 67 (27.91%) participants used bedding material in livestock sheds; however, 173 (72.08%) farmers claimed that they never had used it. Only those farmers (n = 67, 27.91%) who used bedding material were further interviewed to validate which material was commonly used by them and in which season they used them to provide ultimate protection from ticks: 50 (20.83%) claimed that they also used litter leaves, and 17 (7.08%) used paddy straw as bedding material. It was also recorded that 22 (9.16%) farmers used bedding material in the summer season, and 45 (18.75%) farmers used it in the winter season. An investigation showed that famers also used to visit the veterinary centers to receive livestock production inputs (n = 35, 14.58%), to receive medicine for sick animals (n = 58, 24.16%), to receive deworming drugs (n = 52, 21.66%), to receive acaricides (n = 62, 25.83%), and to seek advice on farming practices (n = 33, 13.75%). In addition, 144 (60%) always checked their body for ticks after handling the tick-infested livestock, and 43 (17.9%) farmers sometimes used to check their body for ticks after handling the tick-infested livestock; however, only 53 (22%) farmers mentioned that they never analyze their body.

Other than treating tick infestation, respondents viewed that acaricides could be used as pesticides in the vegetable fields to get rid of insects, lice, mites, and bugs in homes. The three primary reasons people went to livestock centers were: “to receive acaricide”, which was stated by 154 people (64.1%); “to receive medicine”, which was stated by 75 people (20.8%); and “to receive deworming drugs”, which was stated by 11 people (4.5%). Moreover, 100% of the 240 responders said they had used acaricides to get rid of ticks on their livestock. Acaricide usage was indicated by 137 respondents (57.08%) as occurring sometimes, 50 (20.8%) monthly, 41 (17.0% fortnightly), and 12 (5%) weekly, during the peak infestation season, while 230 (95.8%) of the respondents said they used the hand-dressing approach for administering acaricides to host animals, whereas 10 (4.1%) used the hand-spraying method. The respondents were questioned on how long it took for the acaricides to take effect to obtain the fundamental data on the effectiveness of the acaricides utilized: 104 respondents (43.3%) stated that ticks disappeared within a day, 67 (27.9%) within a few hours, 41 (17%) during a few days, and 28 (11.6%) over a week. When asked about the management of the ticks that had fallen off, 133 (55.4%) of the respondents claimed to have done nothing about the management of the ticks that had fallen off, 67 (27.9%) claimed to have flushed them away with water, 24 (10%) claimed to have collected and tossed them into the field, and 16 (6.6%) claimed to have collected and burnt them. After handling tick-infested livestock, 144 respondents (60%) said they “always” examined their bodies for ticks, 43 (17.91%) respondents said they “sometimes” checked, and 53 (22%) said they “never” checked. Similarly, after visiting the forests, 121 (50.4%) farmers claimed that they had “always” checked their physique, 10 (4.1%) farmers reported that they sometimes checked, and nine (3.7%) farmers never checked. 

The respondents stated that they used a combination of manual removal and traditional medicine to manage ticks on livestock when the veterinary centers lacked acaricides. Of these, 102 (42.5%) farmers used manual removal, 66 (27.5%) farmers used Zanthoxylum solution, 55 (22.9%) farmers brushed the animals, nine (3.7 %) farmers applied salt solution, and eight (3.3%) farmers reported doing nothing. A question on whether acaricides may be used for things outside of treating tick infestations was posed to the farmers to ascertain their knowledge of the qualities of acaricides. One hundred twenty-three respondents (42.9%) stated that they were unaware of any additional uses, while 137 (57%) stated that it may be used as either a pesticide (for crops) or an insecticide (for houses). These parameters were evaluated via a questionnaire to obtain a clear analysis of the shelter material used by the farmers in order to prevent animals from tick infestation and tick-borne diseases.

## 4. Discussion

To the best of our knowledge, this was the first study that has been carried out to ascertain livestock producers’ knowledge, attitudes, and practices regarding ticks and TBDs in livestock from selected districts of Sindh, Pakistan. It also provides the perceptions of livestock owners and stakeholders’ knowledge of these limitations across the different AEZs of the study province. However, in past most of the PE studies were focused on pastoral areas to assess the animal health studies throughout the world [25,26]. In most African countries, pastoral communities are mostly found; however, in Pakistan and India, mixed crop–livestock farming practices and production systems are common [27]. 

This study identified the gap in the awareness of livestock owners about TTBDs, and, due to the similarities between education and livestock-rearing practices, this study is likely to reflect the situation in other provinces and neighboring countries. Veterinary and public health issues with ticks and TBDs are widespread, including in Pakistan. TBDs and significant tick infestations are linked to a lower production of milk, meat, and other animal products in many developing nations, as well as animal morbidity and mortality. Ticks spread more diseases than any other species of blood-feeding arthropods on a global scale, harming people and their companion animals, as well as livestock [28]. All research participants included in this study observed ticks except some of them did not see the ticks. Respondents whose daily activities included close contact with livestock, grasslands, and woodlands where ticks are frequently found were interviewed. Although every respondent had seen a tick, more than half of the respondents were unaware of how the livestock become infected with ticks or where the ticks are often located in the environment. Most respondents claimed that ticks were more prevalent during the summer season. Native livestock breeds are typically thought to be quite resistant to ticks, and they may sometimes be raised with little to no tick management because of their natural defenses (immunity) [29], while more than half of the respondents agreed that native breeds were often at risk of obtaining ticks. The views may be inconsistent as, in Pakistan, there are several methods used to raise the native breeds of livestock. In a free-grazing system, the animals spend most of their time in pastures and agriculture land. Similar findings were accorded with research findings from the African countries of Ethiopia and Nigeria, and the Indian state of West Bengal (Bangladesh) [30,31,32,33]. However, several studies also reported contrary statements to our research findings, that insufficient and weakened immune systems in young and elderly livestock were the causes of the increased tick infestation in the young calves and elderly livestock as they were the least cared for, in contrast to the productive adult livestock, which receive the best management treatment [34]. As a result, they could serve as a holding area for persistent environmental tick infestation. Furthermore, our study indicated that the most common tick predilection sites on livestock are the neck and groove regions; however, in another study ([34]), it was revealed that the external genitalia, udder, and perineum were the most infected parts during tick infestation. Our criteria indicated that 52% of the respondents knew enough about ticks to understand their potential role as disease vectors. According to the results of our multiple logistic regression analysis, mastitis, ticks, endoparasites, and viral and bacterial diseases are the more common health problems of animals identified by the respondents, which is in agreement with the ranking score of a study ([17]), which revealed that the most important bovine health and production restrictions for small-scale dairy farmers were endoparasites, clinical mastitis, ticks, hemorrhagic septicemia, reproductive problems, blackleg, and foot-and-mouth disease (FMD). This study also indicated the necessity for basic farmer training courses, as it was discovered that this training had a substantial effect on illness identification as well as the usage of alternative tick management strategies. Other researchers have shown that the extension system for livestock production in communal areas is extremely weak [35,36]. Only a small percentage of farmers reported having had basic farmer training, and the majority of these were above the age of 40. Respondents who use the stall-feeding system for livestock rearing were more likely to have the appropriate knowledge compared to those who used mixed practices. The livestock intensification program encourages the stall-feeding system and had a favorable effect, according to this. Through the sale of milk and other dairy products, monetary revenue is generated under this system, which is primarily focused on improving the health and productivity of livestock. Consequently, a crucial element may be that males have greater possibilities of attending meetings and training sessions organized by the government, which would increase their awareness of ticks.

To collect valuable leaf litter, nearly every home in the nation was granted legal access to a tiny tree grove known as Sokshing in Bhutan [37,38]. Some respondents in our research region claimed to use leaf litter as bedding, particularly in the summer, but this practice also involves inconsistent views because many respondents were compliant with the crop–livestock integration agricultural system that was quickly disappearing. Leaf litter was believed to help ticks thrive. Acaricidal therapy remained one of the most often employed approaches for managing tick populations [29,30]. Almost all the participants in our survey reported using acaricides on livestock and thought that using them was the main way to get rid of ticks. Nearly all of the acaricides were occasionally used, according to the responders (i.e., if it was observed that the animals had heavy tick infestations). These results demonstrate the need for further knowledge on the best ways to employ acaricides and efficient management methods [39,40]. Despite the guidance to respondents to closely follow a recommended dilution rate, there is no system in place to track consumption in the field. 

As a result, the majority of respondents typically avoid applying acaricides during the winter season. As a result, there was a significant tick infestation among livestock, even though the majority of respondents believed they would use acaricides when the winter was finished. Such cultural customs ought to be taken into account as we prepare to develop tick control measures in the future. By conducting in vitro tick immersion experiments with acaricide solutions made according to the manufacturer’s instructions and then analyzing their effects on mortality and female tick egg production, acaricides’ effectiveness on tick susceptibility is evaluated [41,42]. To date, there is no such assessment of the effectiveness of acaricides used in Pakistan. However, watching a tick drop off from an animal’s body after using acaricides might be the basic method of determining the efficacy at the farm level. In our study, most of the participants said the tick drop-off happened within a day, although others said it happened within a few hours, and a few over a few days. However, this information did not provide any meaningful information on the effectiveness of applying acaricides. Respondents either employ manual removal techniques or utilize the seeds of Zanthoxylum armatum DC as a traditional indigenous remedy for tick control when there is a lack of or an unstable availibity of acaricides. Zanthoxylum armatum, sometimes referred to as “Thi-ngye” in Bhutan, is a significant medicinal plant that is found in the subtropical and temperate valleys of the Himalayas, including Bhutan, and has a variety of ethnopharmacological applications [43]. Studies from Pakistan [44] and Brazil [45] have shown that Zanthoxylum spp. were also used as acaricides. Through an adult immersion test with engorged female ticks, the latter discovered the acaricidal capabilities of its essential oil.

The seeds of Zanthoxylum are steeped in water for the night before being administered to the animals [46]. Despite having access to such choices, one-third of the research participants said they did nothing when their livestock center lacked acaricide. This confirmed how crucial it is for the Department of Livestock (DOL) to give respondents a steady supply of acaricides. Other non-chemical tick management techniques, including predators (such as backyard chickens), cleaning the habitat, and rotational grazing [40], may be challenging to implement in Pakistan.

A rising concern among livestock officials was the cross-application of acaricides on the crops in 100% organic agriculture. Half of the respondents said they were unaware of any additional uses, while the other half said it might be used as an insecticide or a pesticide, depending on the situation (pathogens). In the research region, some respondents acknowledged using residual acaricides in their vegetable crops. This research study may have a primary drawback: it was created with a specific place in mind; hence, the results cannot be extrapolated to other regions with different contexts and agricultural systems. Nevertheless, the results do offer helpful data to aid in the creation of educational and outreach initiatives that may be applied even outside the scope of the research. During the survey, the enumerators questioned everyone who was at home, even though the questioning was aimed at household heads. Participatory epidemiological studies are important in addressing TTBDs, but it is crucial to differentiate between various tick species and tick-borne diseases. Keeping this in mind, our study focuses on expanding the research in this direction. We anticipate publishing our findings on the morphological and molecular identification of TTBDs from selected study areas in the upcoming decades. 

## 5. Conclusions

The study identified the gaps in the knowledge and awareness among livestock farmers and stakeholders of Sindh, Pakistan regarding ticks and tick-borne diseases. The results of this study indicating that a focus on educating the respondents about the risks of tick infestations and the measures that can be taken to prevent and control them, such as the proper usage of acaricides or tick-resistant breeds of livestock, can be an effective way to reduce the incidence of tick-borne diseases. Proper livestock management practices, such as maintaining clean and dry living spaces for animals, regular grooming and monitoring for ticks, and prompt treatment of infected animals, can help reduce the incidence of tick-borne diseases. The study identified a lack of access to effective tick control measures, such as acaricides or vaccines, among livestock respondents and herders. Supporting the use of these, measures through either subsidies or education campaigns can be an effective way to reduce the incidence of tick-borne diseases. The study also identified a lack of collaboration and information sharing among different stakeholders involved in tick and tick-borne disease control, such as government agencies, livestock farmers, and veterinarians. Encouraging collaboration and information sharing can help to develop more effective and co-ordinated tick control strategies. Regular monitoring of tick and tick-borne disease incidence among livestock populations can help identify areas and populations that are at higher risk. This can help target interventions, such as education campaigns or tick control measures, in areas where they are most needed.

## Figures and Tables

**Table 1:** Attitude of farmers towards tick control.

Attitude Questions on Likert Scale		
Variables	Opinion	SR (n, %)
Proper use of synthetic acaricides can reduce the cases of tick infestation in livestock.	Strongly disagree	5 (0, 0%)
	Disagree	3 (38, 15.8%)
	No opinion	4 (20, 4.7%)
	Agree	2 (80, 33.3%)
	Strongly agree	1 (102, 42.5%)
The risk of tick infestation can be reduced by always housing livestock in the shed.	Strongly disagree	2 (80, 33.3%)
	Disagree	1 (99, 41.2%)
	No opinion	4 (13, 5.4%)
	Agree	3 (44, 18.3%)
	Strongly agree	5 (4, 1.6%)
Adopting good farm practices can reduce the risk of tick infestation (e.g., regular washing of the floor, regular checking of the animals, avoiding the use of bedding materials, etc.)	Strongly disagree	5 (16, 6.6 %)
	Disagree	4 (18, 7.5%)
	No opinion	2(70, 29.1%)
	Agree	1 (91, 37.9%)
	Strongly agree	3 (45, 18.75%)

n = number of respondents, SR = simple ranking

**Table 2:** Multiple logistic regression analysis for the estimation of the association between explanatory variables and binary outcome.

Variable		Category		Total	Estimated ± S.E	Adjusted OR (95% Cl)	*p*- Values
	**Adequate knowledge**						
**Husbandry** **practice**		**Yes**	**No**				
	Mixed practice	84	44	128	−0.346 ± 0.15	Reference	<0.001
	Stall feeding	61	26	87	1.0387 ± 0.26	2.7 (1.46–4.45)	
	**Intercept**				0.167 ± 0.213		
	**Favorable attitude**	**Yes**	**No**				
**Husbandry** **practice**	Mixed practice	88	52	140		Reference	0.001
	Stall feeding	80	20	100	0.954 ± 0.31	1.8 (1.08–3.43)	
**Common animal health problems**	**Pathogens** **(Favorable attitude)**	**Yes**	**No**	**Total**		Reference	0.001
	Mastitis	88	48	136		1.99 (0.09–2.2)	0.001
	Endoparasitism	104	58	162		0.7 (0.01–1.5)	0.94
	Metabolic diseases	118	108	226		0.4 (0.02–1.25)	0.99
	Bacterial diseases	118	98	216		1.68 (0.05–1.9)	0.2
	Tick infestation	88	28	116		2.5 (1.47–4.06)	<0.05
	Viral diseases	97	31	128		1.88 (1.09–2.9)	0.05
	Plant poisoning	189	45	234		1.2 (1.07–2.78)	>0.1

*p* < 0.05 = significant

## Data Availability

Data available on request.

## Supplementary Materials

The following supporting information can be downloaded at: https://www.mdpi.com/article/10.3390/pathogens12060800/s1, File S1: Questionnaire.
