# Supplementary material for: A Participatory Approach in Assessing the Knowledge, Attitude, and Practices (KAP) of Stakeholders and Livestock Owners about Ticks and Tick-Borne Diseases from Sindh, Pakistan
Source: Pathogens. 2023 Jun 3;12(6):800. doi: 10.3390/pathogens12060800 (PMC10303654; doi:10.3390/pathogens12060800)
Supplement: Supplementary file 1 [file pathogens-12-00800-s001.zip › pathogens-2375085-supplementary.pdf]

S1 Questionnaire

Title: A Participatory Approach in Assessing the Knowledge, Attitude and Practices (KAP) of Stakeholders and Livestock Owners about Ticks and Tick-Borne Diseases from Sindh, Pakistan

Section 1.1: Sociodemographic information of Participant's

1.1.1	Name:	
1.1.2	Age (Years):	
1.1.3	Gender:	1 ☐ Male<br>2 ☐ Female
1.1.4	Level of education:	1 ☐ No school attended<br>2 ☐ Received non-formal education<br>3 ☐ Completed Primary school education<br>4 ☐ High level school<br>5 ☐ Secondary level<br>6 ☐ Graduation
1.1.5	Profession or status	1. ☐ Community member<br>2. ☐ Veterinarian<br>3. ☐ Livestock owner

Section 1.2: Animal husbandry and Managemental strategy

1.2.2	Animal feeding practice at your livestock farm?	1 ☐ Stall feeding<br>2 ☐ All-time free grazing<br>3 ☐ Mixture of all the above
1.2.2	Animal catchment area?	1 ☐ Forested<br>2 ☐ Urban<br>3 ☐ Rural<br>4 ☐ Semi-Urban

Section 2.1: Awareness and knowledge of TTBDs in livestock

2.1.1	Have you ever encountered a tick?	1 ☐ No<br>2 ☐ Yes
2.1.2	If you have encountered a tick, where do you typically find them?	1 ☐ Forest<br>2 ☐ Animals body<br>3 ☐ Pasture land<br>4 ☐ Agriculture land<br>5 ☐ All of the above
2.1.3	In which areas do you believe ticks are mostly found?	1 ☐ Warm places<br>2 ☐ Cold places<br>3 ☐ Both the places
2.1.4	During which season ticks are mostly found on livestock?	1 ☐ Winter<br>2 ☐ Summer<br>3 ☐ Throughout the year

2.1.5	From where do you believe animals acquire ticks?	1 ☐ Grazing land<br>2 ☐ Bedding materials<br>3 ☐ Forest<br>4 ☐ Agriculture land<br>5 ☐ No idea
2.1.6	Which types of livestock breeds do you think are most susceptible to tick infestation?	1 ☐ Indigenous breeds<br>2 ☐ Non- Native breeds<br>3 ☐ No idea
2.1.7	In your opinion which animal breed mostly affected with TTBDs?	1 ☐ Females<br>2 ☐ Old animals<br>3 ☐ Young animals<br>4 ☐ Adults
2.1.8	Most commonly tick infestation site on animal body is?	1 ☐ Neck region<br>2 ☐ Chest and axillae<br>3 ☐ Groin and udder<br>4 ☐ Head region<br>6 ☐ Dewlap<br>7 ☐ Anus and perianal region<br>8 ☐ Others (belly and limbs)
2.1.9	Tick infestation remains always unless it removed?	1 ☐ No<br>2 ☐ Yes<br>3 ☐ Don't know

2.1.12	What are the most devastating effects of ticks on health and production of an animal?<br>You can choose more options<br>.	1 ☐ Bloodsucking<br>2 ☐ Bite wound<br>3 ☐ Anorexia<br>4 ☐ Loss of weight<br>5 ☐ Babesiosis<br>6 ☐ Anaplasmosis<br>7 ☐ Theileriosis<br>8 ☐ Fever<br>9 ☐ Red or brown color urine<br>10 ☐ Hide damage<br>11 ☐ Loss of production<br>12 ☐ Don't know
2.1.13	Animals can get diseases from ticks?	1 ☐ No<br>2 ☐ Yes<br>3 ☐ Don't know
2.1.14	Do you know about any tick-borne disease of animals?	1 ☐ Yes<br>2 ☐ No

Section 2.2: Human being and ticks (A zoonotic perspective)

2.2.1	Did you experience tick bite ever?	1 ☐ No<br>2 ☐ Yes
2.2.2	Which symptoms do you think any person can feel after tick bite?<br>*If you think choose more than one option.*	1 ☐ Fever and headache<br>2 ☐ Irritation with pain<br>3 ☐ Swelling around the bite and rash<br>4 ☐ No symptoms
2.2.3	In your opinion ticks can transmit disease in human beings?	1 ☐ No<br>2 ☐ Yes<br>3 ☐ Don't know

Section 3: Respondents attitudes towards tick control

Attitude Questions on Likert sacale	1	2	3	4	5
3.1 Tick infestations in animals can be reduced by using synthetic acaricides appropriately.	Agree	Strongly agree	Disagree	Strongly disagree	No opinion
3.2 The risk of tick infestation can be avoided by keeping the animals in the shed at all times.	Agree	Strongly agree	Disagree	Strongly disagree	No opinion
3.3 Adopting excellent farm practices (e.g., regular floor washing, regular animal checks, avoiding the use of bedding materials, etc.) helps lower the danger of tick infestation.	Agree	Strongly agree	Disagree	Strongly disagree	No opinion

Section 4: Practices of respondents in livestock farms

4.1	What is the primary reason for raising animal in your household)?	1 ☐ Breeding purpose<br>2 ☐ Sale of animal products<br>3 ☐ Sale of animals to get income<br>4 ☐ Family consumption of products<br>5 ☐ As a source of manure<br>6 ☐ Breeding purpose
4.2	Animal shed type?	1 ☐ Open tethring<br>2 ☐ CGI Sheet and wooden floors.<br>3 ☐ Traditional Shed<br>4 ☐ CGI Sheet and concrete floors.

4.3	Frequency of washing of your concrete livestock farm?	1 ☐ Monthly<br>2 ☐ Daily<br>3 ☐ Weekly<br>4 ☐ Fortnightly<br>5 ☐ Not once
4.4	In your shed do you use bedding material?	1 ☐ No<br>2 ☐ Yes
4.5	If you use bedding material which type you use?	1 ☐ Paddy straw<br>2 ☐ Maize stover<br>3 ☐ Litter leaves<br>4 ☐ Bracken fern
4.6	In which season do you use bedding material?	1 ☐ Winter<br>2 ☐ Summer<br>3 ☐ Throughout the year
4.7	In your opinion which diseases are mostly prevalent in livestock?<br>*Select 3 of the problems*	1 ☐ Endo parasitism<br>2 ☐ Milk fever<br>3 ☐ Mastitis<br>4 ☐ Foot and mouth disease<br>5 ☐ Plant poisoning<br>6 ☐ Bacterial diseases<br>7 ☐ Tick infestation
4.8	Why you visit to veterinary centers and for what purpose you visit to centers?	1 ☐ Livestock products input<br>2 ☐ Medication for sick animal<br>3 ☐ Deworming agents<br>4 ☐ Reciving of acaricides<br>5 ☐ Better farming practice advices.
4.9	In case of tick infestation on your farm how you get rid on this problem?<br>*Respondents can have more than one answer.*	1 ☐ Acaricidal usage<br>2 ☐ Monitor animal and restrict them to outside grazing<br>3 ☐ Rotational grazing<br>4 ☐ Use homemade remedies<br>5 ☐ Manually remove the ticks.

4.10	In what frequency do you use acaricides?	1 ☐ Every week<br>2 ☐ After 15 days<br>3 ☐ In a month<br>4 ☐ Some times
4.11	What is the effective method for acaricidal use?	1 ☐ Spraying with hands<br>2 ☐ Dressing with hands<br>3 ☐ Pour on<br>4 ☐ Mixture of all mentioned practices
4.12	After acaricidal treatment how long does it takes for ticks to fall off?	1 ☐ Within a few hours<br>2 ☐ Within a day<br>3 ☐ Within a few days<br>4 ☐ Within a week.
4.13	When ticks fall from animal body after administering acaricides or ma nually removing or brushing the infested animals. What do you do with ticks?	1 ☐ Let it stay on the ground<br>2 ☐ Collect and throw it in the field<br>3 ☐ Collect and burn<br>4 ☐ Leave them in running water
4.14	When you visit the forest do you check your body after come back?	1 ☐ Always<br>2 ☐ Sometimes<br>3 ☐ Never
4.15	After handling tick infested animals do you check your body?	1 ☐ Never<br>2 ☐ Always<br>3 ☐ Sometimes
4.16	In case of un-availability of acaricides, which method do you used for tick control?	1 ☐ Brush the animal<br>2 ☐ Manually remove<br>3 ☐ With help of kerosene and petroleum products<br>4 ☐ Apply *Zanthoxylum* mixture solution<br>5 ☐ With solution of salt<br>6 ☐ Do nothing

4.17	Where else do you think acaricides can be utilized besides treating tick infestations?	1	☐	To get rid on mites, lice and bugs etc
		2	☐	As pesticides on vegetable fields
		3	☐	No idea
